# Sequential ovulation and fertility of polyoestrus in American black bears (*Ursus americanus*)

**DOI:** 10.1093/conphys/cou051

**Published:** 2014-11-25

**Authors:** Brendan M. Himelright, Jenna M. Moore, Ramona L. Gonzales, Alejandra V. Mendoza, Penny S. Dye, Randall J. Schuett, Barbara S. Durrant, Betsy A. Read, Thomas J. Spady

**Affiliations:** 1 Department of Biological Sciences, College of Science and Mathematics, California State University San Marcos, 333 South Twin Oaks Valley Road, San Marcos, CA 92096-0001, USA; 2 Dakota Hills Veterinary Clinic, Rapid City, SD 57703, USA; 3 Pewaukee Veterinary Service, Pewaukee, WI 53072, USA; 4 San Diego Zoo's Institute for Conservation Research, San Diego Zoo Global, Escondido, CA 92027, USA

**Keywords:** Breeding, embryonic diapause, pseudo-oestrus, recurrent oestrus, superfetation, vulva score

## Abstract

American black bears (*Ursus americanus*) are seasonally polyoestrous and exhibit delayed implantation, which may allow equal and independent fertility of recurrent oestruses of a mating season. We postulated that the luteal inactivity during delayed implantation allows bears to have sequential ovulation during a polyoestrous mating season such that each oestrus of a polyoestrous female will have equivalent fertility, and pregnancy would not preclude subsequent ovulation and superfetation. Controlled mating experiments were conducted on semi-free-ranging female American black bears during three mating seasons, wherein females were bred by different male cohorts in each oestrus. Behavioural observation, vulva score ranking, genetic paternity analysis, gross morphology of ovaries and microscopic morphology of diapaused embryos were used to evaluate the fertility of each subsequent oestrus in polyoestrous females. Oestrus duration, number of successful mounts and median vulva scores were similar between first and subsequent oestruses of the season. Polyoestrus occurred in 81.3% of oestrous females, with a 9.7 ± 5.5 day (mean ± SD) inter-oestrous interval. Sequential ovulation was documented in three polyoestrous females, including one that possessed both a corpus haemorrhagicum and a developed corpus luteum. Among polyoestrous dams, four of nine embryos were conceived in the first oestrus and five of nine in the second oestrus. These results indicate that each oestrus of polyoestrous females is capable of fertility, even if the female is already pregnant from a prior oestrus. Although superfetation was not directly observed in the present study, our results strongly suggest the potential of superfetation in the American black bear and provide novel insight into the complex behavioural and physiological breeding mechanisms of bears. Given that most endangered bear species share similar reproductive traits with American black bears, captive breeding programmes could take advantage of superfetation by mating females with different males at each subsequent oestrus of the season in order to increase the genetic diversity of captive endangered bears.

## Abstract (precis)

In our study, we determined that each of 2-3 estruses (a.k.a. heat) that a female has during a mating season appears to be functional and fertile. Also, being pregnant does not prevent them from coming into heat again that same season. This ability may allow bears to maximize reproductive fitness.

## Introduction

American black bears possess a wide variety of reproductive physiological traits that are likely to serve as strategies to maximize reproductive success, including seasonal polyoestrus (Gonzales *et al*., 2013), delayed implantation (DI) (Wimsatt, 1963), multiple paternity (Schenk and Kovacs, 1995) and, possibly, induced ovulation (Boone *et al*., 2003). Among these reproductive traits, the physiological mechanism of multiple paternity is the least understood and has yet to be studied in bears. Multiple paternity, wherein a single litter is sired by multiple fathers, is known to occur in American black bears (Schenk and Kovacs, 1995) and brown bears (Craighead *et al*., 1995).

In theory, the combination of DI and seasonal polyoestrus should allow American black bears to achieve multiple paternity through a physiological phenomenon known as superfetation. Superfetation is defined as pregnancy occurring while the dam is already pregnant, and is believed to occur when a female becomes impregnated during sequential oestruses of the same mating season (Roellig *et al*., 2011). To date, superfetation has been conclusively documented in only one Carnivora species, the American mink (*Neovison vison*; Shackelford, 1952). While not definitively confirmed, superfetation is also suspected in European badgers (*Meles meles*) based on the fact that they can continue to ovulate following a confirmed pregnancy (Yamaguchi *et al*., 2006). In such species with DI, polyoestrus and superfetation, littermates can be sired by different males during temporally distinct oestruses and ovulations, but are still born at the same time because implantation of embryos is synchronized independent of conception date (Shackelford, 1952; Yamaguchi *et al.*, 2006; Roellig *et al.*, 2011). If these same mechanisms exist in bears, it is plausible to expect that split parturition might result in rare cases when asynchrony of implantation occurs for conceptuses sired in different oestruses. One case of suspected split parturition is documented in wild black bears (Scanlon *et al*., 1998) and one case has been irrefutably confirmed in a captive brown bear litter (Ware *et al*., 2012).

Superfetation is precluded in polyoestrous mammals without DI because females have subsequent oestruses and ovulatory periods per season only if the first oestrus failed to result in conception (Knobil and Neill, 1994; Hafez and Hafez, 2000; Schatten and Constantinescu, 2007); accordingly, only the last oestrus of the season should be conceptive, with no subsequent oestruses occurring in females that are already pregnant. However, this may not be true for polyoestrous species with seasonal DI.

Many polyoestrous species of Carnivora with DI, including bears, American mink and European badgers, have relatively dormant corpora lutea throughout the mating season during the period of DI (Wimsatt, 1963; Canivenc and Bonnin, 1981; Tsubota *et al*., 1987; Tsubota and Kanagawa, 1993). Therefore, the relatively dormant corpora lutea during DI in these species should minimize luteal inhibition of sequential follicular development and ovulation (Knobil and Neill, 1994; Schatten and Constantinescu, 2007; Satué and Gardón, 2013) during the subsequent oestruses of the mating season and permit sequential polyovulation. Sequential polyovulation and sufficient fertile capability of each oestrus within the season of a polyoestrous female should, in turn, permit superfetation.

The purpose of the present descriptive study was to investigate the relative fertility of polyoestrus and to examine the potential underlying physiological mechanisms through which bears achieve multiple paternity. Through a combination of behavioural observation of controlled mating experiments, anatomical and morphological assessment of genitalia, uteri and ovaries, as well as microscopic examination of diapaused embryos and genetic paternity analysis, we sought to compare the relative fertility of each oestrus of polyoestrous females and to determine whether the American black bear is capable of superfetation. We hypothesized that the American black bear is capable of sequential ovulation with independent fertility among each oestrus of the season. Furthermore, we postulated that polyoestrous American black bear females bred in sequential oestruses would exhibit superfetation and that this physiological mechanism may serve to achieve multiple paternity.

## Materials and methods

### Field site, animal disposition and study design

All research, animal handling and care activities were conducted in accordance with approved Institutional Animal Care and Use Committee of California State University San Marcos (CSUSM), Office of Laboratory Animal Welfare assurance 4196. The study bear population consisted of sexually mature, non-lactating female American black bears aged 4–8 years and males aged 5–15 years. Subjects included a total of 16 females (four in 2011, and six each in 2009 and 2013) and 14 males (four in 2009, and each in 2011 and 2013) from a semi-free-ranging population housed in one of two 60-hectare enclosures at a private wildlife reserve in South Dakota. Study females resided in a 305 m × 488 m outdoor enclosure from May to February, and researchers recorded behavioural observations from 27 May to 6 July 2009, from 31 May to 7 July 2011 and from 3 June to 23 July 2013.

As previously described (Gonzales *et al.*, 2013), the onset of behavioural oestrus was determined by using scan sampling with point-in-time observation techniques to observe female sociosexual behavioural changes, and vulva score was used as a proxy to measure physiological oestrus. The duration of physiological oestrus was defined as the number of consecutive days when a female had a vulva score ≥2.5. Consecutive observations of vulva scores below 2.5 indicated the conclusion of an oestrus. When at least one of the six females attained their first and later their second physiological oestruses as determined by vulva score (as described by Gonzales *et al*., 2013), we admitted one cohort of two or three males into the females' enclosure. We kept male cohorts in the pen for 11–19 consecutive days to maximize the probability for successful mating of oestrous females. We used unique sets of two or three males in first and second oestruses of the season during the 2009 (bears M2 and M3 followed by M4 and M5), 2011 (bears M6, M7 and M8 followed by M9 and M10) and 2013 mating seasons (bears M11 and M12 followed by M13, M14 and M15). During the 11–19 day period when males were present in the pen, three observers recorded mating behaviour using focal sampling and all-occurrences recording from 09.00 to 17.00 h daily (624 cumulative hours) in 2009, from 09.00 to 17.30 h daily (535.5 cumulative hours) in 2011 and from 07.30 to 19.30 h daily (1152 cumulative hours) in 2013. Breaks were taken in shifts to minimize missed mating observations, and no observations were collected outside these times. Although it is possible that mating occurred in the evenings when not observed, bears at the study site are not nocturnal or crepuscular (based on 11 years of observing bears at the field site).

After at least one male of the cohort successfully mounted all oestrous females, the males were removed from the pen. The same steps were followed for subsequent oestrous cycles. The minimal period of 10 days without male cohorts present between subsequent oestruses ensured that each oestrus corresponded to a separate oestrous cycle and also that no sperm from the prior set of males could fertilize eggs ovulated during the subsequent oestrous cycles. Black bears do not possess sperm storage glands in their uteri or oviducts (Wimsatt, 1963; Erickson *et al*., 1964); therefore, as with most mammals (Hafez and Hafez, 2000), bear sperm are not anticipated to survive longer than a maximum of 7 days within the female reproductive tract.

### Sample collection and processing

Skin biopsies were collected from males using DNA darts (Pneu-Dart, Williamsport, PA, USA) and a Telinject rifle (Telinject, Agua Dulce, CA, USA) prior to removal from the mating pen. Diapaused embryos were collected after the 2009 and 2011 mating seasons during late and early embryonic diapause (September and July, respectively). Only behavioural and vulva score data were collected in 2013, and the six mated females were allowed to give birth to live cubs. Females were anaesthetized by intramuscular injection of 5–6 mg/kg Telazol™ (1:1 tiletamine:zolazepam; Fort Dodge Animal Health, Overland Park, KS, USA) and 0.8 mg/kg Rompum™ (xylazine; Bayer HealthCare, Shawnee Mission, KS, USA) using a Telinject rifle, and whole blood samples were collected from the jugular vein. Both uteri and ovaries were then removed via a laparoscopically assisted hysterectomy technique. The laparoscopic procedure used a Verres needle, 10 mm Taut trocar/cannula and 5 mm trocar/cannula, with the animal in dorsal recumbancy. Excision was achieved using a 5 mm Harmonic scalpel to cut and seal the ovarian–uterine junction and the uterine body cranial to the cervix. Excised uteri and ovaries were then removed and collected from the 10 females (2009 and 2011) by extracting the trochar and pulling the uterus through the abdominal incision.

Before removing uteri, incisions were made in the ovarian bursa to expose each ovary. The ovarian morphology was carefully examined *in vivo* using a laparoscope, a digital camera and a Sony monitor display. In 2009, the ovaries were also externalized from two of the six females for additional morphological analyses. In 2011, the ovaries were extracted from all four females following removal of uteri. The surface morphology of excised ovaries was then examined before dissection into four equal quarters for morphological analyses of internal structures. The presence of morphological features indicative of ovulation or follicular activity was documented for all ovaries after hysterectomy, including follicles, corpora haemorrhagica and corpora lutea. Corpora lutea become progressively less vascular, have decreased luminal space and shrink as they progress from early to mid-diapause in black bears (Wimsatt, 1963). Accordingly, we used these characteristics to distinguish recent from developed (older) corpora lutea of the same mating season. Corpora lutea were classified as ‘recent’ if they were large, with prominent luminal space and were moderately or highly vascular. Small corpora lutea with reduced luminal space and little or no vascularity were considered ‘developed’ corpora lutea of the season.

The extracted uteri were manually flushed *ex vivo* by thorough rinsing of the lumen with sterile saline solution (0.9 n) into a glass dish. After repeated rinses, horn sections were cut to expose the lumen, and rinsed again to collect any blastocysts not previously removed. All uterine washes were examined under a dissecting microscope, and embryonic size was measured with an ocular micrometer and photographed. Morphology and measurements of the diameter and thickness of the zona pellucida and the diameter of the trophoblast and inner cell mass were recorded to determine the relative developmental stage of diapaused embryos. Skin, blood and embryos were stored in β-mercaptoethanol lysis buffer (QIAGEN Mini-prep kit; QIAGEN Inc., Valencia, CA, USA) for transport (room temperature storage for <48 h) to the laboratory at California State University San Marcos for paternity analyses.

### Determining the conceptive oestrus

DNA was extracted from whole blood of adult females and from diapaused embryos from the 2009 season using the QIAGEN Mini-prep kit. QIAGEN DNeasy Blood and Tissue kits were used to isolate DNA from male skin biopsy samples. Homogenization and mechanical lysis was achieved via mortar and pestle for skin samples and a 20-gauge needle fitted to a sterile syringe for blood and embryos, followed by protease K digestion at 37°C for 22 h.

DNA was then isolated and microsatellites subsequently amplified using PCR. American black bear-specific tetranucleotide primers A107, D11, D103, D112 and D113 (Meredith *et al.*, 2009) were used to amplify polymorphic microsatellite amplicons using Platinum Blue PCR Supermix **(**Invitrogen, Grand Island, NY, USA). The PCR products were separated on 2.5% high sieve agarose horizontal gels at 80 V for 3–5 h or using the Experion capillary electrophoresis system (Bio-Rad, Hercules, CA, USA). Given that the male mating partners were known, paternity was assigned when concordant results were obtained using two or three different polymorphic microsatellite primers.

Given that the embryos were removed during or shortly after the second oestrus in 2011, corresponding to the period of early embryonic diapause, morphological differences in the developmental stage of embryos were informative for determining which oestrus was conceptive. A morula was assumed to correspond to ∼5 days post-conception, while a blastocyst indicated at least 10 days post-conception (Schatten and Constantinescu, 2007). Observed mating dates were then cross-referenced to determine the precise post-conception age of embryos.

### Data analysis

As the study design was observational and descriptive, we used medians with 95% confidence intervals for data comparisons, unless otherwise stated, to avoid statistical over-interpretation and biases caused by assumptions of normal distributions. Student's paired *t*-test was used to detect differences in the number of times that females were successfully mounted in their first and subsequent oestruses. The Wilcoxon rank sum test was used to detect differences in the order of the conceptive oestrus of polyoestrous females. The Kendall coefficient of concordance and Pearson correlation coefficients were used to determine inter-observer reliability for field observations of animal behaviour and vulva scores (Martin and Bateson, 2007). All statistical analyses were performed using Minitab v16.0 (Minitab Inc., State College, PA, USA) and α < 0.05 level of significance.

## Results

All of the 16 females included in the study attained physiological oestrus, and 13 of the 16 (81.3%) also had polyoestrus (Supplementary Table S1). One female from 2011 was considered polyoestrous by vulva scores and direct anatomical observations of ovaries that showed distinct sequential ovulations, but did not mate in either oestrus. The inter-oestrous interval of physiological oestrus, measured as consecutive days during which the vulva score was <2.5, averaged 9.7 ± 5.5 days (mean ± SD; range 3–21 days) in the 13 polyoestrous females.

**Figure 1:**
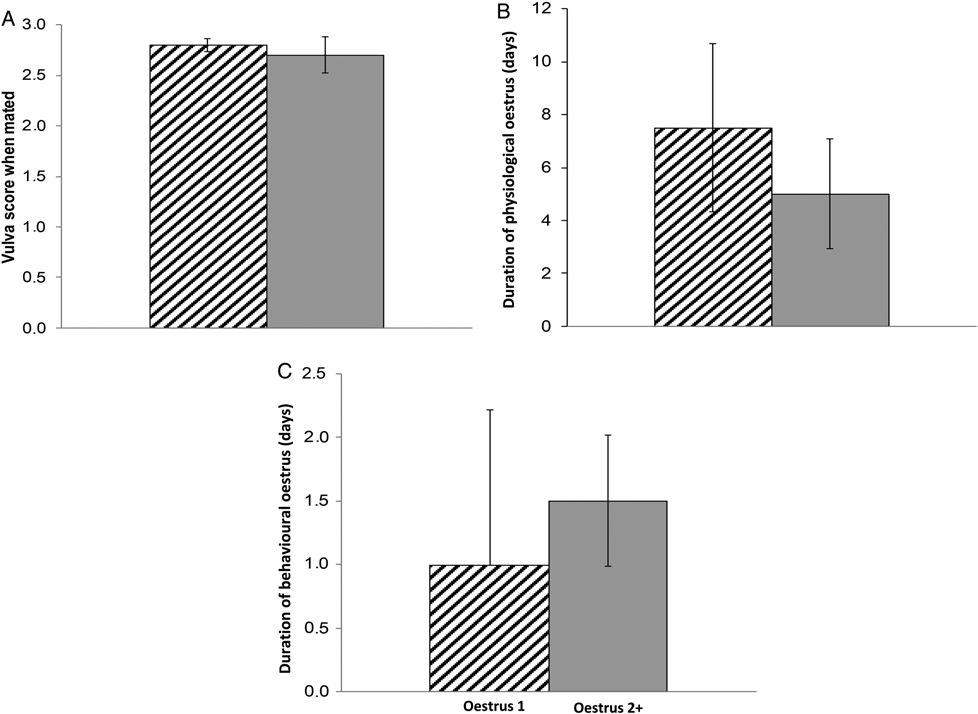
(**A**) Median vulva score of females when mated during the first (hatched bar; *n* = 35) compared with subsequent oestruses (grey bar; *n* = 9). For each median, the *n* values indicate the cumulative number of days that females were observed mating in the indicated oestrus. (**B**) Median duration of physiological oestrus, as proxied by vulva score 2.5/3 or greater, during the first (hatched bar; *n* = 16 oestruses) compared with subsequent oestruses (grey bar; *n* = 13 oestruses). (**C**) Median duration of behavioural oestrus during the first (hatched bar; *n* = 13 oestruses) compared with subsequent oestruses (grey bar; *n* = 8 oestruses). Error bars represent the 95% confidence intervals.

There was no discernible difference between the median vulva score on the day mated (Fig. 1A; range 2.3–3.0 and 2.0–2.9 for first oestrus and subsequent oestruses, respectively) or the median duration of the first physiological oestrus and any subsequent oestruses (Fig. 1B; range 1–20 days for first oestrus and 1–13 days for subsequent oestruses).

**Figure 2:**
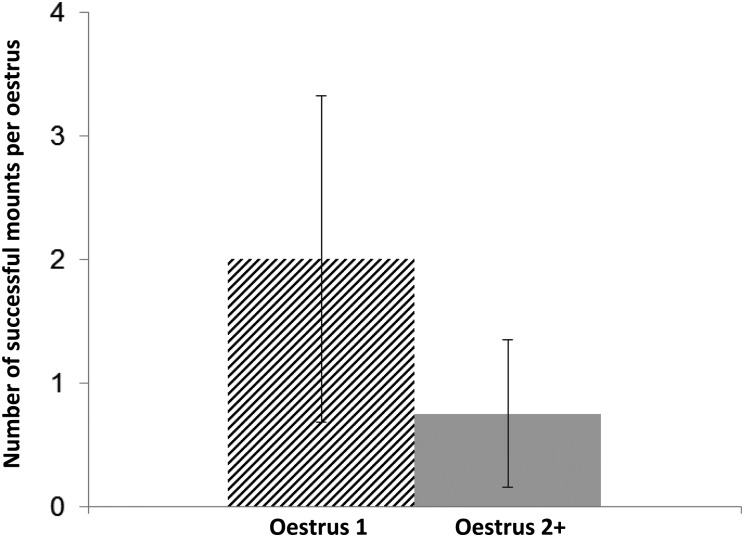
Mean number of successful mounts occurring during the first physiological oestrus (hatched bar; *n* = 12 oestruses) and subsequent oestruses (grey bar; *n* = 12 oestruses) in polyoestrous females. Error bars represent the 95% confidence intervals. For each polyoestrous female, repeated measures of the same female were paired by oestrus order (first, subsequent) for analyses of the population using Student's paired *t*-test.

First and sequential oestruses were similar in both the median duration of behavioural oestrus, as measured by the number of consecutive days of successful mounts (Fig. 1C; range 1–7 days for first oestrus and 1–3 days for subsequent oestruses), and the mean number of times that a polyoestrous female was successfully mounted during each oestrus (Fig. 2; *t* = 1.67, d.f. = 23, *P* = 0.124, Student's paired *t*-test).

Three of the four mated polyoestrous females examined during early diapause (8–9 July 2011) exhibited sequential ovulation. The fourth (F4-11) had a developed corpus luteum on each ovary. Two females had both recent and developed corpora lutea within the same pairs of ovaries, and one female possessed a corpus haemorrhagicum, enlarged fimbria and a bursa distended with viscous fluid in one ovary and a developed corpus luteum in the contralateral ovary. In contrast, pairs of ovaries examined in five mated polyoestrous females during late diapause (25–26 September 2009) each possessed two or more developed corpora lutea, but no corpora haemorrhagica or recent corpora lutea.

Of the nine embryos retrieved from polyoestrus females after the 2009 and during the 2011 mating seasons, four were sired during the first oestrus, while the remaining five were sired during the second oestrus (Table 1). When assessed during early diapause, embryos represented developmental stages ranging from compact morulas (estimated 5 days post-conception) to early or mid-stage blastocysts (26–28 days). By late diapause, five of six embryos were developed blastocysts and one had even achieved blastocoel expansion. The estimated embryo age (from conception date) was moderately correlated with trophoblast diameter (*r* = 0.64, *P* = 0.048, Pearson correlation), but embryos of the same age and litter varied in diameter by as much as 1200 μm by late diapause (Table 1).

**Table 1:** Temporal comparison of conceptive oestruses among polyoestrous females as assessed during diapause

Dam	Embryo ID	Conceptive oestrus	Embryo age (days)	Trophoblast diameter (μm)	Stage of embryo
F2-09	E1	First	111	1400	Blastocyst
F2-09	E2	First	111	2600	expanded Blastocyst
F4-09	E3	Second	93	800	Blastocyst
F4-09	E4	Second	93	760	Blastocyst
F5-09	E5	Second	91	600	Blastocyst
F5-09	E6	Second	91	480	Blastocyst
F1-11	E7	Second	5	200	Compact morula
F4-11	E8	First	26	500	Early blastocyst
F6-11	E9	First	28	460	Early blastocyst

The order of the oestrus of conception (first or second) was determined by paternity testing of embryos collected in 2009 using PCR and microsatellite primers. For 2011 embryo samples, conceptive oestrus was determined by comparison of mating records with microscopic examination of the approximate developmental stage of embryos. Embryonic age was estimated by subtracting the latest date when females were observed mating the sire during the conceptive oestrus from the date of diapaused embryo collection.

Of the six late diapause embryos whose paternity was identified genetically, four were sired during an oestrus in which more than one male successfully mounted the dam (Supplementary Table S2). Both males M2 and M3 successfully mated the polyoestrous female F2-9, but the DNA quality of M2 was too poor for proper PCR detection of microsatellite alleles. However, M2 was deduced to be the sire of F2-9's litter by a process of elimination, because the *Uam107* and *UamD113* alleles of M3 did not match those of the embryos. The two-embryo litter of F3-9 was excluded from all analyses because genetic paternity assessment was not clearly distinguishable. Although the potential for superfetation is strongly suggested by our observation of sequential ovulation and the fertility of sequential oestrus periods before or during delayed implantation, neither multiple paternity nor superfetation was observed in the present study.

## Discussion

The present study supports the conclusion that each oestrus exhibited by polyoestrous female black bears during a mating season is physiologically and behaviourally functional and is capable of resulting in conception. We also demonstrate sequential ovulation and independence of the oestrous cycle on pregnancy status in black bears. Collectively, these data suggest that American black bears are capable of superfetation. These findings may also hold true for other polyoestrous bear species owing to their similarity in reproductive physiology, most notably the mechanisms of DI and polyoestrus (Spady *et al*., 2007; Steyaert *et al*. 2011).

Among polyoestrous females, the first and second oestruses were physiologically indistinguishable (Fig. 1A and B). In addition, the similarity between first and second oestruses in the behavioural measures of the duration of oestrus (Fig. 1C) and the relative sexual interest of both males and females based on successful mounts in both oestruses (Fig. 2) indicate that each oestrus of a polyoestrous female is behaviourally equivalent. Although the modestly shorter median duration of behavioural oestrus and modestly greater number of mounts in the first compared with the second oestrus were not significant (overlapping 95% confidence intervals), it is possible that differences might have been detected with a larger sample size. Also, although we deem it unlikely given the diurnal activity of bears at the study site, some matings may have occurred outside the daily observation sessions. Overall, however, these data support the conclusion that each oestrus of a polyoestrous female is functional and fertile.

The discovery of both a recent and previous ovulation through the presence of a corpus haemorrhagicum and corpora lutea in the ovaries of a pregnant female (F6-11) establishes that American black bears are capable of sequential ovulation, because the ovulations occurred at temporally distinct oestruses. This conclusion is further supported by the presence of both recent and developed (older) corpora lutea in two polyoestrous females in July. Even though five polyoestrous females in September exhibited only developed corpora lutea in their ovaries, it is feasible that the corpora lutea formed in sequential ovulations, but had both already developed into mature corpora lutea by the time they were examined during late diapause (immediately prior to corpus luteum reactivation). Although multiple ovulations during a single oestrus have been previously documented in black bears (Erickson *et al*., 1964), sequential ovulations have not.

One of the strongest lines of evidence indicating that each oestrus of polyoestrus is capable of fertility is that pregnancy of polyoestrous females had a similar likelihood of occurring in the first oestrus compared with the subsequent oestruses of the season (Table 1). Therefore, active pregnancy during a prior oestrus did not preclude subsequent ovulation and polyoestrus.

Our findings contradict prior assumptions in early bear studies, which suggested that only one of multiple oestruses is fertile during the mating season (Meyer-Holzapfel, 1957). Oestruses early in the season were considered to be pseudo-oestruses, which were presumed to be subfertile and probably anovulatory. These subfertile cycles were proposed to serve the purpose of priming the reproductive system for a subsequent fertile oestrous cycle (Meyer-Holzapfel, 1957). As a result of these early presumptions on the mechanisms of polyoestrus in bears and their assumed similarity to polyoestrus of well-documented domestic polyoestrous species without DI (Knobil and Neill, 1994; Hafez and Hafez, 2000; Schatten and Constantinescu, 2007), the potential for fertile recurrent oestrus, sequential ovulation and superfetation in bears had never been tested empirically.

The frequent occurrence (in 13 of 16 females) of polyoestrus and the fact that it can result in sequential ovulation independent of pregnancy status raises important questions about the evolutionary significance of polyoestrus. The high frequency of occurrence and equivalent fertile capability of polyoestrus may serve to maximize mating opportunities and reproductive success of black bears. In combination, these polyoestrous characteristics should increase the chances of superfetation. Moreover, our findings are comparable to what Yamaguchi *et al.* (2006) found in the European badger (*M. meles*), wherein superfetation is postulated because pregnant females continue to have fertile oestruses during embryonic diapause.

Although we did not observe superfetation in the present study, our data support the hypothesis that American black bears are capable of superfetation. The fertility of sequential oestruses suggests there are no physiological, behavioural or anatomical barriers preventing superfetation. Our findings suggest that DI may have evolved in bears not only to detach conception from parturition in order to increase dam and neonate fitness (Sandell, 1990), but also to detach oestrous cycles from pregnancy in order to maximize conception opportunities via superfetation.

By maximizing the chances of multiple paternity, superfetation may provide many evolutionary benefits related to the increase of reproductive fitness. For instance, increased offspring diversity may be used as a mechanism to limit inbreeding depression (Sillero-Zubiri *et al*., 1996; Jennions and Petrie, 2000) or as a bet-hedging strategy to minimize fitness variation of the offspring (Watson, 1991; Jennions and Petrie, 2000). By increasing the number of perceived fit sires, there is decreased risk that overall litter fitness will be low. In brown bears, multiple paternity has been proposed as a probable female reproductive strategy to increase paternity confusion in order to avoid infanticide by males (Steyaert *et al*., 2011). Other possible evolutionary benefits of multiple paternity include an increase in ‘good genes’ or ‘compatible genes’ (Foerster *et al.*, 2003; Wolff and Macdonald, 2004). Superfetation would increase the effects of any of these direct evolutionary benefits to the offspring by increasing the frequency of multiple paternity.

Elucidation of the mechanisms of superfetation and multipaternity could improve the ability of wildlife managers to minimize loss of genetic diversity in endangered bear species, including small populations with fragmented habitats. In addition, superfetation could be applied to improve husbandry of endangered bear species in zoos. New artificial insemination protocols devised to inseminate a female in different oestruses with sperm from different males or naturally mating a female with different males in each subsequent oestrus may provide viable new approaches to increase genetic diversity.

## Supplementary material

Supplementary material is available at *Conservation Physiology* online.

## Funding

This work was supported by the California State University Program for Education and Research in Biotechnology Faculty Student Seed grant [to T.J.S.]. Support for R.L.G. and A.V.M. was provided by the National Institute for General Medical Sciences Research Initiative for Scientific Enhancement [grant number GM064783].

## Supplementary Material

Supplementary Data
