# Supplementary material for: Sequential ovulation and fertility of polyoestrus in American black bears (Ursus americanus)
Source: Conserv Physiol. 2014 Nov 25;2(1):cou051. doi: 10.1093/conphys/cou051 (PMC4732479; doi:10.1093/conphys/cou051)
Supplement: Supplementary Data [file supp_2_1_cou051__index.html]

Supplementary Data 

# Sequential ovulation and fertility of polyoestrus in American black bears (*Ursus americanus*)

## Supplementary Data

Supplementary Data

**Files in this Data Supplement:**

- Supplementary Table 1 - doc file
- Supplementary Table 2 - doc file
