# Supplementary material for: Sequential ovulation and fertility of polyoestrus in American black bears (Ursus americanus)
Source: Conserv Physiol. 2014 Nov 25;2(1):cou051. doi: 10.1093/conphys/cou051 (PMC4732479; doi:10.1093/conphys/cou051)
Supplement: Supplementary Data [file supp_cou051_cou051supp_table1.doc]

**SupplementaryTable 1:** Frequency of recurring estrus during the 2009-2013 mating seasons.

| **Season** | **# Females** | **# of Estrous Females** | **Monoestrus** | **Polyestrus** |
| --- | --- | --- | --- | --- |
| 2009 | 6 | 6 | 1 | 5 |
| 2011 | 4 | 4 | 0 | 4 |
| 2013 | 6 | 6 | 2 | 4 |
| Total | 16 | 16 | 3 | 13 |
| % of total |  | 100 | 18.7 | 81.3 |
