# Supplementary material for: Sequential ovulation and fertility of polyoestrus in American black bears (Ursus americanus)
Source: Conserv Physiol. 2014 Nov 25;2(1):cou051. doi: 10.1093/conphys/cou051 (PMC4732479; doi:10.1093/conphys/cou051)
Supplement: Supplementary Data [file supp_cou051_cou051supp_table2.doc]

**Supplemental Table 2:** Genetic paternity analyses from diapaused embryos collected in late diapause.

| **Litter** | **Sample** | ***UamA107*** | ***UamD11*** | ***UamD113*** |
| --- | --- | --- | --- | --- |
| **1** | F2-9 | 157 |  | 174; 218 |
|  | E1 | 157 |  | 174 |
|  | E2 | 157 |  | 174 |
|  | **M2*** | **?** |  | **?** |
|  | M3 | 146; 162 |  | 165 |
| **2** | F4-9 |  | 226 | 200 |
|  | E3 |  | 226 | 200 |
|  | E4 |  | 226 | 200 |
|  | **M5*** |  | **226** | **200** |
|  | M3 |  | 245; 255 | 165 |
| **3** | F5-9 | 157; 196 | 180 |  |
|  | E5 | 157; 196 | 180 |  |
|  | E6 | 157; 209 | 180 |  |
|  | **M4*** | **157; 209** | **180; 200** |  |
|  | M5 | 214 | 226 |  |

Column headings *UamA107*, *UamD11* and *UamD113* indicate the specific tetranucleotide microsatellite loci used as target primers, and the values under those columns represent the length in base pairs of the sample allele(s). Question marks represent unobtainable DNA, while blanks represent an unused allele for a litter. Two additional embyros (from F3-09) were excluded from analyses due to complexity in resolving paternity. Bolded and asterisked males are sires.
